# Experiences and coping strategies of parents living with adolescents misusing *nyaope* substance

**DOI:** 10.4102/hsag.v27i0.2042

**Published:** 2022-11-08

**Authors:** Jan Masombuka, Faith Mathibela

**Affiliations:** 1 Department of Social Work, Faculty of Human Sciences, University of South Africa, Pretoria, South Africa

**Keywords:** adolescents, coping strategies, experiences, *nyaope*, misusing, parents

## Abstract

**Background:**

*Nyaope* is a South African substance whose usage continues to escalate among young people. Regrettably, the negative effects of this scourge impact not only the misusers but also their parents who unavoidably have to endure the problems associated with their adolescents’ dependence.

**Aim:**

The study sought to explore the experiences and coping strategies of parents living with adolescents who misuse *nyaope*.

**Setting:**

The study was undertaken in Soshanguve township, situated in the north of the city of Tshwane in the Gauteng province of South Africa.

**Methods:**

Using a qualitative approach, the study adopted dimensions of both explorative and descriptive designs to develop an in-depth understanding of experiences and coping strategies of parents living with adolescents who misuse *nyaope* in Soshanguve Township. Individual face-to-face semistructured interviews through purposive sampling were conducted by the researchers to collect data from eight parents of such adolescents.

**Results:**

Three themes emerged, namely parents’ experiences of how they detected the onset of the adolescents’ misuse of *nyaope*, effects of adolescents’ misuse of *nyaope* on parents and family as a whole and parents’ coping strategies in dealing with the adolescents.

**Conclusion:**

From the shared stories, it is evident that parents are overly concerned about the extent of damage cause by adolescents’ misuse of *nyaope* within the family system.

**Contribution:**

This study will enable scholars in the field of substance misuse to conduct further research on the phenomenon and generate new knowledge that will contribute towards formulating future policies and implementing new intervention strategies.

## Introduction

The misuse of *nyaope* amongst South African young people continues to gain popularity in Soshanguve township (Mokwena 2016:138). *Nyaope* is a South African substance and is commonly misused by young black Africans, who are mainly from disadvantaged backgrounds (Meel & Essop 2018:585). The National Drug Master Plan (2019–2024:8) describes *nyaope* as a cocktail mixture of drugs with a high concentration of heroin and cannabis (Department of Social Development 2020). *Nyaope* is typical to South Africa; therefore, there are no data that can be used to compare how this substance is misused anywhere else in the world (Motsoeneng 2018:6). Accordingly, Mokwena and Huma (2014:353–354) noted that although *nyaope* is reported to have emerged from city of Tshwane; soon after, it quickly spread to other townships. The actual ingredients of this substance are numerous and varied. Some researchers suggest that it contains antiretroviral drugs, cocaine and rat poison (Mathibela 2017:5; Motsoeneng 2018:1).

*Nyaope* bears different names depending on the region. For instance, it is known as *wunga* or *whoonga* and ‘sugars’ in KwaZulu-Natal and *ungah* in the Western Cape, while in Limpopo and Mpumalanga it is called ‘pinch’ and in Johannesburg *kataza* (Mokwena & Huma 2014:354). Since the emergence of this substance in the Tshwane metropolitan area during the early 2000s, its selling price of R30 per joint has not increased substantially (Mokwena & Huma 2014:354; Motsoeneng 2018:1; Radebe 2017:20). To ensure that prices remain the same, dealers opt to decrease the quantities per joint so that misusers can afford and continue to support the *nyaope* market (Meel & Essop 2018:585; Mokwena & Huma 2014:354).

From the onset, smoking has been the most common method of misusing *nyaope* (Masombuka 2013:2; Mokwena 2016:1). However, recently, there is a new trend of injecting *nyaope* directly into one’s body (Meel & Essop 2018:585; Phokedi 2018:13). This process is called ‘Bluetooth’. ‘Bluetooth’ involves sharing *nyaope* by drawing blood from a misuser who is already high and injecting it to a ‘buddy’ in order to share their high (Radebe 2017:21). Scientifically, this method raises questions regarding its effectiveness in getting a ‘buddy’ high. However, what is evident is that this practice is adding more health risks to the *nyaope* fraternity (Phokedi 2018:15). Misusers of *nyaope* perform this process under unhygienic conditions where many of them use the same syringe multiple times. This results in misusers developing, amongst other health conditions, septicaemia. They normally present with swollen veins which, in most instances, develop into gangrene (Mahopo 2018). Consequently, since February 2017, Dr George Mukhari Academic Hospital in the north of the city of Tshwane has operated on more than 50 misusers of *nyaope* because of the aftereffects of the ‘Bluetooth’ method (Mahopo 2018).

A report by the South African Community Epidemiology Network on Drug Use (SACENDU) reveals that *nyaope* use is more prevalent in men, with 83% being admitted for treatment in the Gauteng province between January 2019 and July 2019, as compared to their female counterparts who account for only 17% of the total (Dada et al. 2019:17). Between January 2018 and June 2018, the average age for a *nyaope* misuser was 31 years (Dada et al. 2019:19). Accordingly, *nyaope* is perceived as an integral part of black communities in South Africa, and it contributes to a deprived social environment which just gets worse with time (Mokwena & Huma 2014:360).

According to Groenewald (2018:1564), the misuse of substances is widespread, and it negatively impacts the psychosocial well-being of parents of misusers. A study by Mathibela (2017:89–90) gives an example of a mother from the Cape Flats in the Western Cape province of South Africa who endured years of abuse by her substance misusing son. Finally, she retaliated by strangling him as a way of ending her misery. It is therefore important to understand the experiences and coping strategies of parents in relation to the adolescents’ misuse of substances. The outcomes can contribute to the development of appropriate support interventions to enable parents to cope effectively with the difficulties they experience (Groenewald 2018:1565). In this study, ‘parent’ refers to the father, mother, adoptive parents or grandparents living in the same household with an adolescent misusing *nyaope*, while ‘adolescent’ refers to a misuser of *nyaope* living with the parent(s).

It is overwhelming and worrisome for parents to learn that their adolescent child is misusing a hazardous substance such as *nyaope*. Despite the difficulty and burden of managing misusers of *nyaope*, there is inadequate research that focuses on parents as caregivers who assume responsibility to deal with the problem associated with the adolescents’ behaviour (Radebe 2017:vi). Furthermore, parents of adolescents misusing *nyaope* are not well informed about how to manage the situation (Motsoeneng 2018:48). In recognition of these constraints, this study sought to become the voice of parents in expressing their experiences and coping strategies of living with adolescents misusing *nyaope*.

The study is framed in the family systems approach. In line with this approach, a family is an organism that is more than the sum of its parts, which is constantly interacting with its environment, both affecting and being affected by it, that has boundaries between the family and the environment and within the family itself and that is capable of growth and change (Shulman 2016:292). Putting it differently, Kirst-Ashman (2017:22) postulated that the family system is a set of components that are related to each other with the purpose of making a functional whole. From the above, it can be implied that adolescents misusing *nyaope* and their parents are subsystems that tie together a family as a system. Therefore, when an adolescent misuses *nyaope*, the other parts of the family system become affected, and eventually the entire system might collapse.

Given the evidence from previous studies conducted in relation to adolescents misusing *nyaope*, it is apparent that parents are severely affected by the situation (Motsoeneng 2018:iii; Nkosi 2017:i). However, there is a dearth of evidence regarding the experiences and coping strategies of parents living with adolescents misusing *nyaope* (Masombuka & Qalinge 2020:53; Radebe 2017:vi). Therefore, this study aims at exploring these experiences and coping strategies.

## Research methods and designs

### Design

A qualitative research approach with an explorative and descriptive research design was employed to provide the knowledge and insight into the phenomenon under investigation. The use of the qualitative approach enabled the researchers to collect data by giving participants time to describe their experiences and give opinions in relation to the phenomenon being studied. The researchers are qualified social workers with respective postgraduate qualifications and are experts in the field of qualitative research. In addition, both researchers are registered with the South African Council for Social Service Professions (SACSSP) and thus adhere to a professional code of ethics.

### Setting

The study was conducted in Soshanguve township, which is one of the communities known to have the highest prevalence of *nyaope* misuse (Mokwena 2016). In addition, antidrug abuse programmes, if any, in Soshanguve are known to be ineffective and unsuccessful despite the overwhelming prevalence of *nyaope* misuse, the lack of recreational amenities and the high youth unemployment rate (Charlton, Negota & Mistry 2019:21). It is also noteworthy that all participants chose to be interviewed at the researchers’ offices.

### Study population and sampling strategy

The population for this study consisted of parents living with adolescents misusing *nyaope* in Soshanguve Township. Due to the nature of the population, time and financial constraints, it was impractical for the researchers to study everybody in the targeted population; hence, there was a need for a sample. In this study, the sample provided responses that reached saturation after the eighth interview. The researchers knew that saturation point was reached when the repetition of information became evident. However, because of the stigma attached to substance misuse, parents of adolescents misusing substances are a difficult population to reach (Choate 2015:463). Therefore, purposive sampling was appropriate in guiding the researchers to select relevant participants for the study (Creswell 2013:167). Subsequently, the sample size of eight parents living with adolescents misusing *nyaope* was recruited from a private practice. Permission to conduct the study was sought from the private practice manager as a gatekeeper. Consequently, the participants were identified from the previous caseload at the private practice with the gatekeeper’s assistance. In addition, the gatekeeper was mobilised for support to telephonically inform identified participants about the study. Thereafter, face-to-face meetings were scheduled with the respective parents where the researchers were officially introduced and presented the purpose of the study. To ensure that the study was completely voluntary, the gatekeeper as an independent professional person facilitated the process of signing the informed consent forms. All signed informed consent forms were handed over to the researchers, who commenced the actual data collection process.

To ensure that the identified participants provided relevant data for the study, the researchers relied on the information provided by the gatekeeper, and it was later verified through biological questions during data collection. During data collection, the researchers explained the sample inclusion criteria, which entail only parents who were living in the same household with adolescents misusing *nyaope*; had previously reached out for professional assistance at private practice; were conversant in English, Setswana and/or IsiZulu; and resided in Soshanguve township.

### Data collection

Individual face-to-face semistructured interviews, informed by an interview guide, were used by researchers as a data collection method. Rosetto (2014:483) stated that the main purpose of the semistructured interview in qualitative research is to gather information and facts. Therefore, in this study, all interviews were audio-recorded with the participants’ consent. Each semistructured interview lasted between 40 min and 60 min. In addition, participants’ preferred language of interview was English. Furthermore, observations and open-ended questions were employed by the researchers to explore experiences and coping strategies of parents living with adolescents misusing *nyaope*. Thereafter, the audio-recorded interviews were transcribed by the researchers and the independent coder for an in-depth understanding of the responses. It is noteworthy that all interviews were conducted by the researchers.

### Data analysis

Tesch’s framework guided the process of qualitative data analysis (Creswell 2014:196). The framework was deemed appropriate for this study as it allowed the researchers to analyse responses related to the experiences and coping strategies of the participants. In analysing data, the researchers worked with an independent coder, who, like them, employed Tesch’s framework for qualitative data analysis.

### Measures of trustworthiness

Guba’s model for assessing the trustworthiness of qualitative data guided the process (Krefting 1991:215). The credibility of the study was enhanced by using various interviewing techniques such as listening, observation, probing, restating and summarising. To achieve transferability, a comprehensive description of the research methodology employed was provided. In ensuring the dependability of this study, the independent coder and the researchers independently coded the data and subsequently held consensus discussion on the themes and subthemes, which were presented as research findings. Conformability of the study was achieved by ensuring that the findings represent the experiences and coping strategies of participants and not the values and characteristics of the researchers.

### Ethical considerations

The Health Research Ethics Committee (HREC) at North-West University granted the ethical permission to embark on this study, and the ethics number NWU-00025-18-S1 was allocated. The principle of informed consent was observed by ensuring that participants were fully informed that participation was purely voluntary, and they had the option to exit the study at whatever time they felt uncomfortable. In addition, confidentiality and anonymity were observed by ensuring that the participants’ identity and data furnished is not disclosed to those outside the research fraternity. In addition, pseudonyms were used as opposed to real names of the participants. The audio-recordings were stored in a locked office, and only the researchers had access to them.

It is noteworthy that because of the sensitivity of the phenomenon under investigation, a free debriefing session or any kind of counselling that the participants might need after data collection was arranged. However, none of the participants in this study needed to undergo debriefing sessions.

## Research findings

The biographical data on each participant’s gender, occupation and race, as well as the adolescents’ age and gender, are presented in Table 1.

**TABLE 1:** Biographical data of the participants.

Participants	Gender	Occupation	Race	Adolescent participant’s age	Adolescent participant’s gender
Participant 1	Female	Employed	Black African	17	Male
Participant 2	Female	Employed	Black African	18	Male
Participant 3	Female	Employed	Black African	15	Male
Participant 4	Female	Unemployed	Black African	16	Male
Participant 5	Female	Employed	Black African	16	Male
Participant 6	Male	Employed	Black African	15	Male
Participant 7	Female	Employed	Black African	14	Male
Participant 8	Male	Retired	Black African	18	Male

*Source:* Masombuka, J., 2020, Parents as an under-utilised resource for the substance dependency service to their youth: Developing a model for social work practitioners, Thesis for the Doctor of Philosophy in Social Work, at North-West University

The biographical profile of participants reflects that most of them were mothers living in the same household with adolescents misusing *nyaope*. This correlates with the findings by Smith, Estefan and Caine (2018:512), who reported that because of the pressure on women to be ‘good mothers’, they are compelled to conform to the expected role of being nurturers within the family system. Only two participants were men (fathers) living in the same household with adolescents misusing *nyaope*. Along the same lines, findings from a study by Kalam and Mthembu (2018:474) revealed that fathers’ involvement in the adolescents’ well-being is imperative and therefore a key contributor in reducing the risk of substance misuse by adolescents.

In relation to the occupation of participants, most of them (six) were employed, one was unemployed, while the other lived a retired life. On the employment issue, Kalam and Mthembu (2018:474) attested that parents’ employment conditions push them away from home most of the time, leaving their adolescents unattended and at the risk of engaging in substance misuse. Concerning the race, all participants were black Africans. This correlates with the report by Statistics South Africa which estimates that black Africans account for 81% of the South African population (Statistics South Africa 2019:8). Meanwhile, the finding by Mokwena and Huma (2014:361) highlighted that the trends of misusing *nyaope* follow the lines of race, and in this case, it is predominant amongst black Africans.

The ages of the participants’ adolescents misusing *nyaope* ranged from 14 to 18 years. This is in concurrence with a finding by Hoeck and Van Hal (2012:1), who reported that although substance misuse occurs in all age groups, young people are the most vulnerable population. Regarding the gender of adolescents misusing *nyaope* whose parents were interviewed, all participants reported that they were male. This correlates with the finding by Hoeck and Van Hal (2012:9), which showed that the misuse of substances was a primary problem for boys and men.

The discussion of themes and subthemes that were generated from the interviews with parents living with adolescents misusing *nyaope* is presented in Table 2.

**TABLE 2:** Themes and subthemes.

Themes	Subthemes
Theme 1: Parents’ experiences of how they detected the onset of the adolescents’ misuse of *nyaope*	How parents detected the onset of adolescents’ misuse of *nyaope* through school Parents being victims of theft by the adolescents Neglect of hygiene by the adolescents
Theme 2: Effects of the adolescents’ misuse of *nyaope* on parents and family as a whole	Conflictual relations amongst family members Disengagement with the family
Theme 3: Parents’ coping strategies in dealing with the adolescents	Giving adolescents money to buy *nyaope* Reaching out to the church

*Source:* Masombuka, J., 2020, Parents as an under-utilised resource for the substance dependency service to their youth: Developing a model for social work practitioners, Thesis for the Doctor of Philosophy in Social Work, at North-West University

## Theme 1: Parents’ experiences of how they detected the onset of the adolescents’ misuse of *nyaope*

Participants were requested to state their experiences of detecting the onset of the adolescents’ misuse of *nyaope*. Three subthemes were generated under this theme.

### Subtheme 1: How parents detected the onset of adolescents’ misuse of *nyaope* through school

Participants reported that schools played key roles in alerting them about the adolescents’ misuse of *nyaope*. They did not have prior knowledge about the adolescents’ misuse of *nyaope* until the school made them aware. The following reports capture how participants detected the onset of adolescents’ misuse of *nyaope* through school:

‘I knew nothing about the misuse of *nyaope* until the school complained to me that he was playing truant and was caught in possession of substances by the teacher.’ (Participant 4, 40 years, mother)

‘It was only after the school informed me that he had been caught in possession of substances that I learnt about my child’s problem.’ (Participant 7, 59 years, mother)

The above findings resonate with Choate’s (2015:467) assertion that it is common for parents to be unaware of the adolescents’ substance misuse. In addition, Waini (2015:82) pointed out that school is a main source of information for parents in making them aware of the problem. Related to the study, Manu and Maluleke (2017:15) noted that illegal substances in South Africa are so readily available and cheap to the extent that learners can afford to buy them. This enormously contributes to a high rate of substance misuse by school-going adolescents, especially those located within urban and peri-urban communities of the country. Paying attention to the issue, Jacobs and Slabbert (2019:230) found that the selling and distribution of illegal substances is prevalent within the South African school environment.

### Subtheme 2: Parents being victims of theft by the adolescents

When it comes to being victims of theft, participants shared that the strange behavioural pattern of stealing from them was a revelation of the adolescents’ misuse of *nyaope*. Since the aforementioned was an unfamiliar experience for participants, they did not know how to respond to the criminal behavioural pattern of the adolescents. Consequently, the experience of being victims of theft by the adolescents was overwhelming and costly for parents, resulting in them feeling vulnerable in their own homes. The comments that follow were extracted from the transcripts in relation to parents being victimised because of theft by the adolescents:

‘He steals my money, cutlery, clothes and cell phones. I noticed also that the Holy Bible is missing. I do not understand the desperation that makes him to even steal a Holy Bible. That makes me realise that something is wrong with my son.’ (Participant 1, 30 years, mother)

‘The list of items that he steals at home is endless and when I confront him, he denies it until his brother caught him with evidence.’ (Participant 8, 66 years, father)

Related to the current study, Mahlangu and Geyer (2018:337) found that misusing *nyaope* pushes misusers to steal valuable items from significant others. In addition, findings by Mthembi, Mwenesongole and Cole (2018:115) revealed that the misuse of *nyaope* in South Africa has resulted in young men resorting to criminal activities which include stealing anything valuable that they can lay their hands on to feed their habit. Inevitably, the parent–adolescent relationship is severely constrained, because parents cannot trust the adolescents in their homes. As a precautionary measure, parents make sure that they lock the houses when they are away from home in order to protect their property. In addition, they become hypervigilant when they are around the house with these adolescents (Ngantweni 2018:41).

### Subtheme 3: Neglect of hygiene by the adolescents

Furthermore, participants reported that they also detected the onset of misuse of *nyaope* when the adolescents displayed poor personal hygiene. For participants, it was disappointing to observe the extreme neglect of hygiene. The following comments were extracted from the transcripts in relation to how participants detected the onset of misuse of *nyaope* through the neglect of hygiene practices by the adolescents:

‘I noticed that he wore the same clothes for days. I also noticed that his room was disorganised and smelling.’ (Participant 6, 59 years, father)

‘It became impossible for him to take a bath on daily basis, unless he was being pestered.’ (Participant 2, 35 years, mother)

The above responses correlate with the finding that misusers of *nyaope* are easily identifiable by their poor personal hygiene (Mokwena 2016:138). For parents, it is disheartening to discover that *nyaope* destroyed the interest of the adolescents in personal hygiene. They realised that this substance took precedence over the adolescents’ personal cleanliness. In the same vein, a study about the experiences of *nyaope* misusers in the three provinces of South Africa revealed that *nyaope* took over their lives; it controlled them in that when they became dependent misusers, getting the next fix was always a priority (Mokwena & Huma 2014:352). The term ‘fix’ is the slang used by the misusers of *nyaope* to describe the required dosage needed to achieve the desired effect (Motsoeneng 2018:33).

## Theme 2: Effects of the adolescents’ misuse of *nyaope* on parents and family as a whole

The second theme that emerged from the interview data relates to effects of the adolescents’ misuse of *nyaope* on parents and family as a whole. The following subthemes were generated under the theme.

### Subtheme 1: Conflictual relations amongst family members

Participants lamented that the adolescents’ misuse of *nyaope* is the main trigger of conflicts amongst the family members. Furthermore, participants explained that the pilfering behaviour of the adolescents sparked anger and frustration amongst the family members. As a defence mechanism, family members fight with the adolescents in order to try to stop the continuous disruption within the family system. The following observations captured the essence of conflictual family relations as described by participants:

‘There is no harmony at home. Daily, he is either involved in a fight with his siblings or father over the missing items.’ (Participant 5, 54 years, mother)

‘He is the centre of conflict in our family. There is always conflict between him and his siblings and between me and him, as well as between me and my husband over his behaviour.’ (Participant 3, 39 years, mother)

The above findings from participants resonate with the assertion that it is common for the substance misusers to cause poor relationships within the family through their behaviour. Furthermore, it is common for misusers to have conflict-laden relationships with siblings and their extended family (Ngantweni 2018:42). Groenewald (2018:1568) also asserted that it is common for substance misusers to become belligerent towards their mothers and other family members. In addition, Radebe (2017:128) opined that the interaction with family members grows less while the conflict grows more in families, as a result of the behaviour of adolescents misusing *nyaope*. Motsoeneng (2018:29) concurred by revealing that often adolescents disrupt smooth functioning and create new and unfavourable dynamics in their families.

### Subtheme 2: Disengagement of the family

According to the participants, the adolescents’ misuse of *nyaope* causes severe disruption and disharmony within their families. The continuous disharmony caused by the behaviour of the adolescents compromised the normal functioning of the entire family system. As a result, the stability of the entire family system was on the verge of collapsing as the adolescents and other family members spent less time at home. This experience was overwhelming for participants, as it resulted in the disengagement of the family. Thereafter, the adolescents and other family members spent more time away from home, while those at home locked themselves in their rooms. The following comments captured the essence of disengagement of family relations as described by participants:

‘Other members disengage from the family activities by locking themselves in their rooms, while he spends days away from home.’ (Participant 7, 59 years, mother)

‘The house is always empty and lonely because family members have withdrawn themselves from the family by spending less time at home.’ (Participant 8, 66 years, father)

These views expressed by the participants correlate with the finding that in order to avoid conflict at home, the adolescents misusing *nyaope* resort to disengaging from their families to spend quality time with other misusers (Nkosi 2017:50). In the study by Mokwena and Huma (2014:359), misusers of *nyaope* acknowledged tearing their families apart because of overindulgence in the substance. In addition, they admit that they are an embarrassment to their families and have put the parents in an agonising position. Notably, the tumultuous behaviour of substance misusers is the main cause of the breakdown of mother–adolescent relationship. When this happens, the relationship that was described as ‘good’ is exchanged for one characterised by mistrust and a lack of warmth (Groenewald 2018:1570). Motsoeneng (2018:47) expounded that other family members resort to obtaining protection orders in an attempt to protect themselves and stop engagement with the adolescent misusing *nyaope*.

## Theme 3: Parents’ coping strategies in dealing with the adolescents

When participants were interviewed, it was discovered that they had developed coping strategies in dealing with the behaviour of the adolescents misusing *nyaope*, which range from giving adolescents money to buy *nyaope* and reaching out to a church.

### Subtheme 1: Giving adolescents money to buy *nyaope*

Participants in the study reported that they give money to the adolescents in order to buy *nyaope*, as a coping strategy to prevent further problems. For participants, offering financial support to purchase *nyaope* was a way of protecting the adolescents from stealing from community members, which would in turn put their family at the risk of being attacked. Participants expressed the following sentiments:

‘I give him money on a daily basis to buy *nyaope* because I am scared that if I do not, he might go out and steal from others, which might cause more problems for him and my family.’ (Participant 6, 40 years, mother)

‘I am scared if I do not give him money to buy *nyaope*, he will create more problems for me; hence, I opt to give it.’ (Participant 2, 35 years, mother)

Consistent with these findings, Hoeck and Van Hal (2012:7) opined that it is customary for some parents to enable misusers by providing them with money to buy substances. Choate (2015:468) also found that some parents pay off the drug debts of their adolescents as a way of coping and protecting the family. However, Kirst-Ashman (2017:469) cautioned that such practice by family members has dire consequences, as it enables the misusers to take less responsibility for their actions. In addition, Mahlangu and Geyer (2018:229) warned family members against enabling misusers of *nyaope* to buy substances by offering financial support.

### Subtheme 2: Reaching out to church

Participants indicated that the church provided support to enable them to cope with the problems emanating from the misuse of *nyaope* by the adolescents. For participants, a church provides a readily available coping strategy as well as spiritual healing.

‘I always go to church because if I am there, I get comfort and a sense of hope is instilled in me about the difficulty that I am facing.’ (Participant 4, 40 years, mother)

‘The support from the church is overwhelming, and without it, I would not be able to overcome everything.’ (Participant 3, 39 years, mother)

In line with the findings, Waini (2015:144), recommended that in South Africa, there is a need to find ways in which the church can be integrated as an institution of support for parents of adolescents misusing substances. In addition, there is also a need to promote the role of religion as a coping strategy for parents of adolescents misusing substances. In concurrence, Mathibela and Skhosana (2021:5) confirmed that parents of adolescents who misuse substances rely on the support and therapy given at their local churches by their pastors. Furthermore, the church as a tool that guides society to godly lives has an instrumental role to play in the fight against substance misuse (Kosgei, Mutua & Pam 2021:33).

## Discussion

The study aimed to explore the experiences and coping strategies of parents living with adolescents misusing *nyaope*. Evidence from the data and literature reveals that *nyaope* is already a cause for concern to parents of adolescents who misuse this substance. The study confirmed that school plays a key role in alerting parents about the adolescents’ misuse of *nyaope*. In this regard, Radebe (2017:132) confirmed that it is common for parents to discover the adolescents’ misuse of *nyaope* while in middle school. Sadly, the study also showed that parents are victims of theft by the adolescents. The devastating experience of being victimised by the adolescents misusing *nyaope* is incalculable for both parents and their families. These findings resonate with Groenewald’s (2018:1569) assertion that parents became victims of monetary theft resulting from the adolescents’ substance misuse problem.

Regarding the matter of neglected personal hygiene, it is a concern for parents to witness how *nyaope* misuse transforms the adolescents from young people who maintain good personal hygiene to strangers with a lack of interest in personal cleanliness. It was evident to parents that misuse of *nyaope* takes precedence over the personal hygiene of the adolescents. Related to the current study, Nkosi (2017:41) cited that family members are stressed about the state of poor personal hygiene of adolescents misusing *nyaope*.

Furthermore, the study revealed that the behaviour of adolescents misusing *nyaope* has dire consequences for the affected families, as they cannot function as units. In line with the finding, Schultz and Alpaslan (2016:91) pointed out that substance misuse is not an individual problem only but also affects the family system. Furthermore, Radebe (2017:128) argued that the adolescents’ misuse of *nyaope* often throws the family into turmoil because of conflicts. Accordingly, parents always find themselves in the middle of friction between these adolescents and other family members. This often poses a challenge as their attempts to maintain peace and normal family functioning are consistently jeopardised by the negative behaviour of the adolescents. In addition, because of theft by the adolescents, parents and other family members are at risk of crime at home.

Regarding coping strategies, parents opt to give the adolescents money to buy substances as a way of saving both the family and the misuser. In concurrence, Mathibela and Skhosana (2021:5) observed that the practice of giving adolescents money to buy substances is the parents’ way of coping and protecting the entire family. Furthermore, parents acknowledge that the support from the church, pastor and prayers enables them to cope with the challenge.

Regarding the study strengths and limitations, it is noteworthy that parents of adolescents misusing *nyaope* are often overlooked and barely feature in research. Accordingly, the strength of the study lies in its focus on parents, who unavoidably endure the problems associated with the adolescents misusing *nyaope*. Unfortunately, the study is limited only to parents living with adolescents misusing *nyaope* in Soshanguve township, which is one of the communities in South Africa known to have the highest cases of *nyaope* misuse. Therefore, the findings cannot be generalised as it is likely that a similar study undertaken in a different community might yield different results.

## Conclusions

The findings of this study indicate that school plays a significant role in sensitising parents about the adolescents’ misuse of *nyaope*. Based on the parents’ experiences and coping strategies, it is concluded that collaboration with schools goes a long way in making parents aware of the adolescents’ misuse of *nyaope*. Regarding the aspect of parents being victims of theft, the researchers concluded that parents of adolescents misusing *nyaope* need psychosocial enhancement and protection from criminal activities by the adolescents.

It is also concluded that the adolescents’ loss of interest in personal cleanliness is distressing to parents. From the shared stories, it is evident that parents are genuinely concerned about the extent of damage caused by the adolescents’ misuse of *nyaope* within the family system. The researchers concluded that the behaviour of the adolescents misusing *nyaope* leads to conflict and disintegration of the family as a unit and has the potential to collapse the entire family system. Furthermore, it is concluded that parents, as the centre and backbone of a family system, require support to save the entire family system from collapsing as a result of their adolescents’ misuse of *nyaope*.

In addition, the researchers concluded that the adolescents’ misuse of *nyaope* inevitably leads to a breakdown in family relationships as family members spend more time away from home. In response to parents giving misusers money to purchase *nyaope*, it is concluded that there is a need for programmes to educate and support parents of adolescents misusing *nyaope*. Finally, it is concluded that religious institutions such as church have a significant role to play in supporting parents of adolescents. There is also a need for collaboration between social workers, parents, families, educators, church leaders and law enforcement agencies in combating adolescents’ misuse of *nyaope*.
